# BMI Affects the Relationship between Long Chain N-3 Polyunsaturated Fatty Acid Intake and Stroke Risk: a Meta-Analysis

**DOI:** 10.1038/srep14161

**Published:** 2015-09-15

**Authors:** Pengfei Cheng, Wen Huang, Shunjie Bai, Yu Wu, Jia Yu, Xiaofeng Zhu, Zhiguo Qi, Weihua Shao, Peng Xie

**Affiliations:** 1 Department of Neurology, the First Affiliated Hospital of Chongqing Medical University, Chongqing, 400016, China; 2 Chongqing Key Laboratory of Neurobiology, Chongqing, 400016, China; 3 Institute of Neuroscience and the Collaborative Innovation Center for Brain Science, Chongqing Medical University, Chongqing, China; 4 Department of Histology and Embryology, Jiamusi University, Jiamusi, Heilongjiang Province, 154002, China; 5 Department of Neurology, Xinqiao Hospital, Third Military Medical University, Chongqing 400037, China; 6 Key Laboratory of Medical Diagnostics, Ministry of Education; 7 Department of Laboratory Medicine, Chongqing Medical University, Chongqing 400016, China; 8 Institute of Neuroscience, Jiamusi University, Jiamusi, Heilongjiang Province, 154002, China; 9 Department of Neurology, Jiamusi University, Jiamusi, Heilongjiang Province, 154002, China; 10 Department of Respiratory Medicine, the First Affiliated Hospital, Chongqing Medical University, Chongqing, 400016, China

## Abstract

We performed a meta-analysis to clarify the relationship between long chain n-3 polyunsaturated fatty acid (PUFA) intake and stroke risk. Relevant studies were identified by searching online databases through May 2015. Log relative risks (RRs) of the highest versus the lowest for cohort studies were weighed by the inverse variance method to obtain pooled RRs. Fourteen prospective cohort studies including 514,483 individuals and 9,065 strokes were included. The pooled RR of overall stroke risk for long chain n-3 PUFA intake was 0.87 [95% confidence interval (CI), 0.79–0.95]. Stratification analysis showed that higher long chain n-3 PUFAs intake was associated with reduced fatal stroke risk (RR = 0.84; 95% CI, 0.73–0.97), reduced stroke risk for BMI < 24 (RR = 0.86; 95% CI, 0.75–0.98) and reduced stroke risk for females (RR = 0.81; 95% CI, 0.71–0.92), but was not associated with stroke risk for either BMI ≥ 24 or men. This meta-analysis reveals that higher long chain n-3 PUFA intake is inversely associated with risk of stroke morbidity and mortality with BMI and sex as key factors influencing this risk. Individuals should be encouraged to manage their body weight while increasing their intake of long chain n-3 PUFAs.

Cerebrovascular diseases primarily include ischemic stroke (i.e., thrombotic infarction or lacunar infarction), hemorrhagic stroke (i.e., subarachnoid or intraparenchymal hemorrhage induced by hypertension or arteriosclerosis), and transient ischemic attack (TIA; i.e., transient ischemia induced by arteriosclerosis or microthrombi). In 2010, stroke-related deaths totaled 5.9 million^1^ with stroke survivors experiencing higher frequencies of disability^2^. Therefore, developing a better understanding of the factors that drive stroke risk can improve these adverse outcomes.

Long-chain n-3 PUFAs, including eicosapentaenoic acid (EPA, 20:5 ω −3), docosapentaenoic acid (DPA, 22:5 ω −3), and docosahexaenoic acid (DHA, 22:6 ω −3), which are almost exclusively derived from marine sources^3^, have been shown to enhance atherosclerotic plaque stability while lowering blood pressure and blood triglyceride levels^4^,^5^. Although one recent meta-analysis^6^ revealed that long chain n-3 PUFA intake shows no association with coronary disease risk, one previous meta-analysis^7^ found no significant association between long-chain n-3 PUFA intake and total stroke risk but did detect a significant inverse association with ischemic stroke. Another meta-analysis^8^ demonstrated an inverse association between long chain n-3 PUFA intake and risk of cerebrovascular accident.

Giving this conflicting evidence and four recent prospective studies^9^,^10^,^11^,^12^, the role of long chain n-3 PUFA intake in stroke risk remains uncertain. Moreover, the influence of body mass index (BMI) upon the effects of long chain n-3 PUFA intake on stroke risk remains unknown. Thus, here we performed a meta-analysis of prospective studies to clarify the association between long chain n-3 PUFA intake and the risk of stroke morbidity and mortality.

## Results

### Literature search results

**Figure 1:**
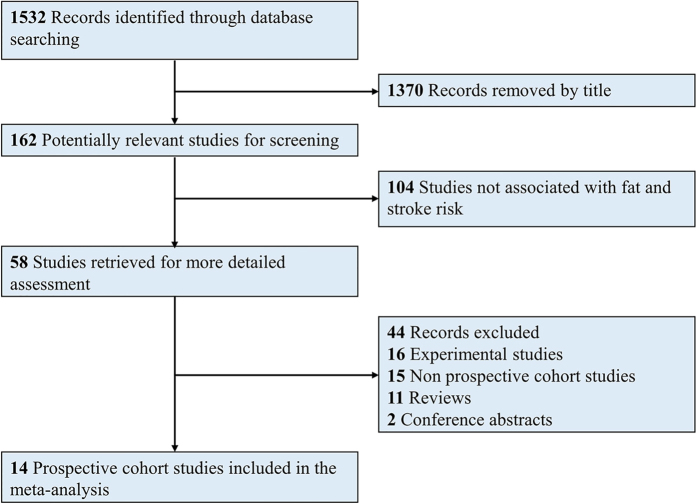
Flow chart of study selection.

The detailed flowchart of study selection is shown in Fig. 1. A total of 1532 records were initially identified; of these, 1370 records were excluded by title/abstract screening. Of the 162 potentially relevant records, 104 records were excluded because they were not associated with stroke risk, and 44 additional records were further excluded for the following reasons: 16 were experimental studies, 15 were non-prospective studies, 11 were reviews, and two were conference abstracts. Thus, 14 prospective studies^9^,^10^,^11^,^12^,^13^,^14^,^15^,^16^,^17^,^18^,^19^,^20^,^21^,^22^ were finally included in this meta-analysis.

### Study characteristics

A summary of the characteristics of the included studies is shown in Table 1. The fourteen prospective studies^9^,^10^,^11^,^12^,^13^,^14^,^15^,^16^,^17^,^18^,^19^,^20^,^21^,^22^ were published from 1995–2014 and included 514,483 individuals ranging from 34 to 84 years of age and a total of 9,065 stroke events. The total number of subjects included in each study ranged from 2,710 to 134,296 subjects, and the total number of stroke events included in each study ranged from 69 to 1,680. The average follow-up duration ranged from 4 to 28 years. Eleven studies^9^,^10^,^12^,^13^,^14^,^16^,^18^,^19^,^20^,^21^,^22^ used food frequency questionnaire (FFQ), two studies^15^,^17^ used 24-hour recall, and one study^11^ used both FFQ and 2-hour recall for long chain n-3 PUFA intake assessment. Study quality was assessed using the Newcastle-Ottawa Scale (NOS)^23^; seven studies^9^,^10^,^12^,^15^,^16^,^19^,^22^ were classified as high-quality (>8 stars), while the remaining seven studies^11^,^13^,^14^,^17^,^18^,^20^,^21^ were deemed low-quality (≤8 stars).

### Primary outcome

**Figure 2:**
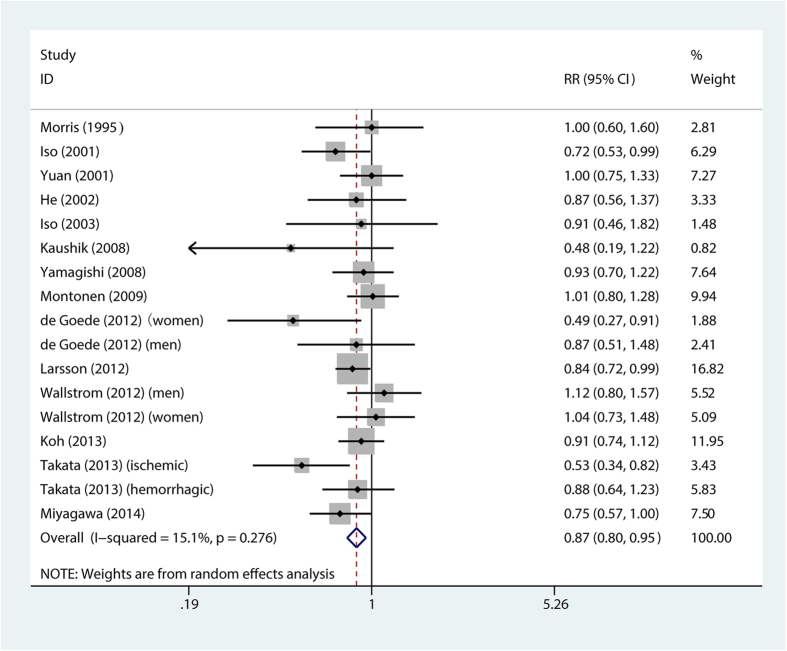
Forest plot of relative risk for long chain n-3 PUFA intake and overall stroke risk. RR, relative risk.

Higher long chain n-3 PUFA intake was associated with reduced overall stroke risk [relative risk (RR) = 0.87; 95% confidence interval (CI), 0.79–0.95] (Fig. 2).

### Secondary outcome

**Figure 3:**
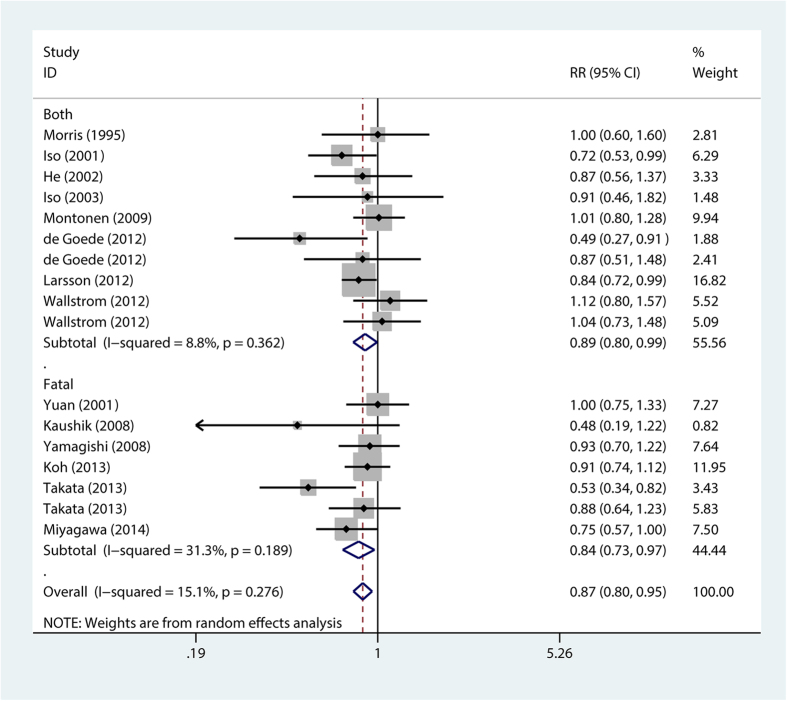
Forest plot of relative risk for long chain n-3 PUFA intake and fatal stroke risk. RR, relative risk. Both analysis includes mixed fatal and non-fatal stroke risk.

**Figure 4:**
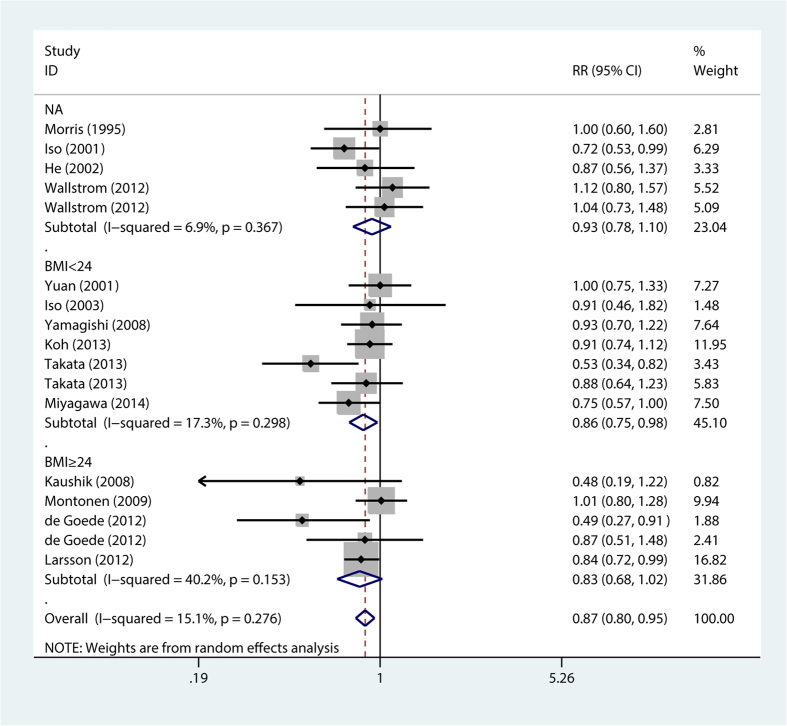
Forest plot of relative risk for BMI effects in long chain n-3 PUFA intake and stroke risk. NA, not available; RR, relative risk; BMI, body mass index.

**Figure 5:**
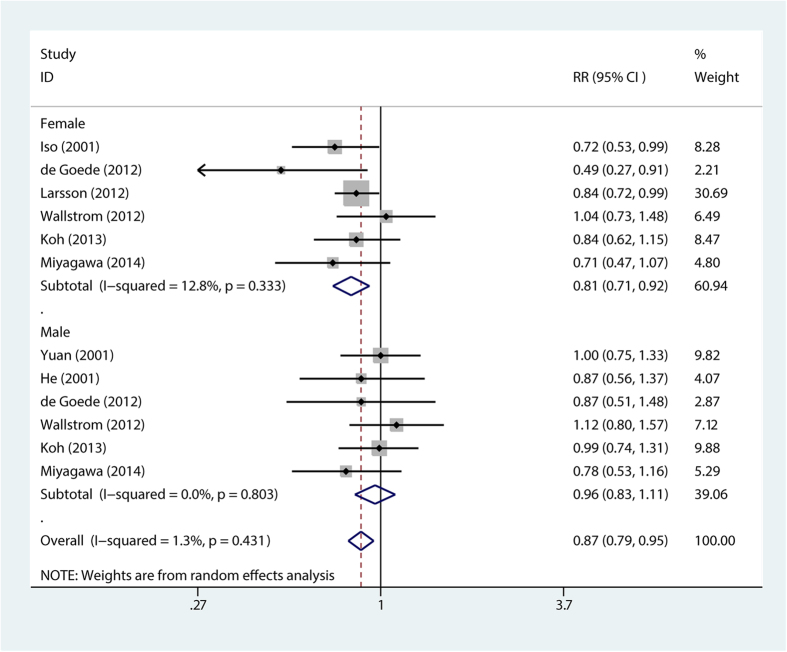
Forest plot of relative risk for long chain n-3 PUFA intake and stroke risk for sex subgroups. RR, relative risk.

The stratification analysis showed that higher long chain n-3 PUFA intake was associated with reduced fatal stroke risk (RR = 0.84; 95% CI, 0.73–0.97) (Fig. 3), reduced stroke risk for BMI < 24 (RR = 0.86; 95% CI, 0.75–0.98) (Fig. 4), reduced stroke risk for follow-up duration ≤14 years (RR = 0.87; 95% CI, 0.78–0.98) and >14 years (RR = 0.87; 95% CI, 0.76–0.99), reduced stroke risk for non-East Asians (RR = 0.86; 95% CI, 0.75–0.97) and East Asians (RR = 0.86; 95% CI, 0.75–0.98), reduced stroke risk for females (RR = 0.81; 95% CI, 0.71–0.92) (Fig. 5), reduced stroke risk for ischemic stroke (RR = 0.87; 95% CI, 0.76–0.99) and hemorrhagic stroke (RR = 0.82; 95% CI, 0.68–0.99), reduced stroke risk for maximum multivariates (RR = 0.88; 95% CI, 0.82–0.96), and reduced stroke risk for quality score ≤8 (RR = 0.79; 95% CI, 0.66–0.94) and >8 (RR = 0.9; 95% CI, 0.82–0.98). However, higher long chain n-3 PUFA intake was not significantly associated with stroke risk for BMI ≥ 24 (RR = 0.83; 95% CI, 0.68–1.02) and men (RR = 0.96; 95% CI, 0.84–1.11) (Table 2).

### Meta-regression analysis

Although we used meta-regression analysis to explore the potential sources of heterogeneity, we did not determine the sources of heterogeneity.

### Sensitivity analysis

**Figure 6:**
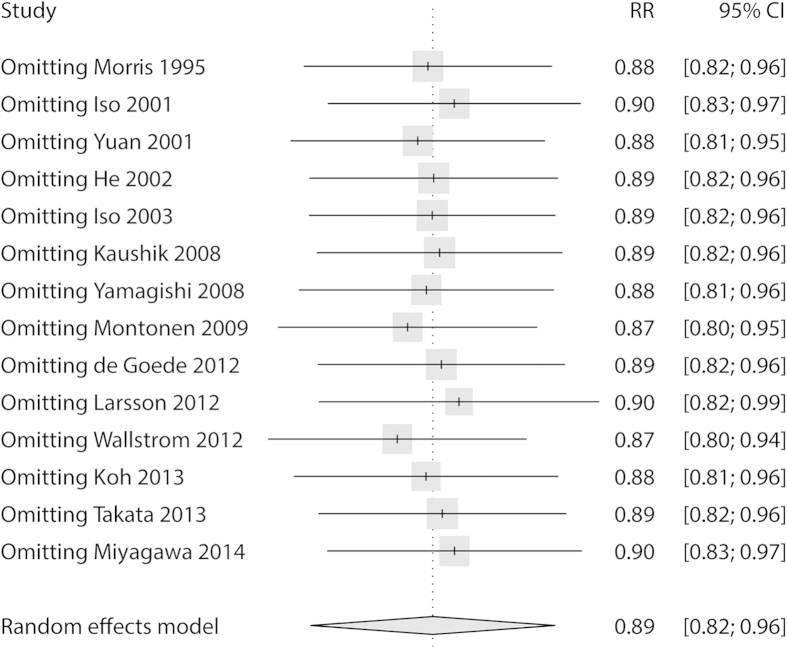
Sensitivity analysis of relative risk for long chain n-3 PUFA intake and stroke risk. The results remained persistent after applying the leave-one-out method.

Sensitivity analysis demonstrated that the relationship between higher long chain n-3 PUFA intake and reduction of stroke risk remained persistent after applying the leave-one-out method (Fig. 6).

### Publication bias

**Figure 7:**
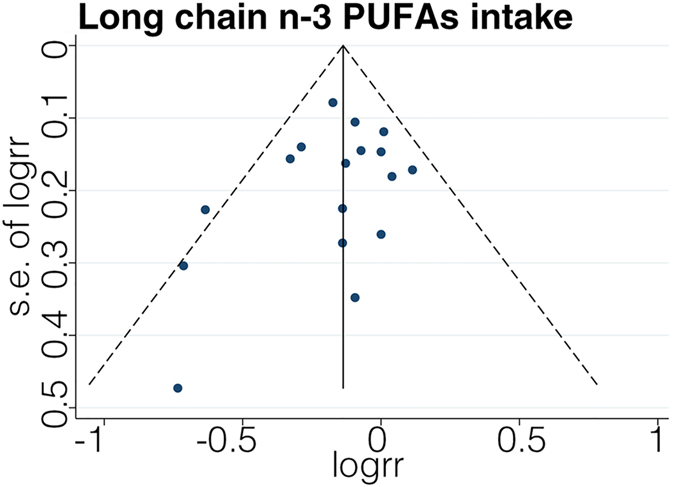
Funnel plot showing association of long chain n-3 PUFA intake with stroke risk.

The funnel plot was symmetrical by visual inspection (Fig. 7), and no significant publication bias was statistically detected by Egger’s test (*p* = 0.28).

## Discussion

This meta-analysis consisting of 14 prospective studies of 514,483 individuals and 9,065 stroke events reveals that higher long chain n-3 PUFA intake is associated with a reduced overall stroke risk, which confirms the findings from a previous meta-analysis^8^. In contrast, four recent prospective studies found inconsistent findings regarding the relationship between long chain n-3 PUFA intake and stroke risk. For instance, one study^9^ found no association between long chain n-3 PUFA intake and stroke risk in either men or women, and another study^10^ found no association between long chain n-3 PUFA intake and stroke mortality. In contrast, another study^11^ demonstrated an inverse association between long chain n-3 PUFA intake and ischemic (but not hemorrhagic) stroke risk, while the most recent prospective study^12^ reported a significant reduction in stroke mortality for only the highest quartile of long chain n-3 PUFA intake.

The mechanism(s) by which higher long chain n-3 PUFA consumption contributes to decreased stroke risk remain unknown. Some lines of evidence^4^,^5^ show that dietary long chain n-3 PUFA enhances the stability of atherosclerotic plaques, lowers blood pressure, decreased blood triglyceride concentrations, decreases inflammation, and improves vascular function. There is evidence^24^ demonstrating that consumption of processed meats is associated with higher incidence of coronary heart disease and diabetes mellitus, while a recent meta-analysis study^25^ indicates that consumption of fresh or processed red meat as well as total red meat is positively associated with increased risk of total stroke and ischemic stroke.

Red meat is a source of heme iron. Higher iron mediates damage to tissues by catalyzing the production of reactive oxygen species (ROS), which leads to lipid peroxidation, protein modification, and DNA damage^26^,^27^,^28^,^29^. One meta-analysis^30^ showed that higher intake of heme iron is associated with an increased risk of cardiovascular disease. Moreover, red meat is also a source of saturated fat and cholesterol; accordingly, a higher intake of saturated fat from meat or high absorption of cholesterol is associated with greater risk of cardiovascular disease^31^,^32^. Moreover, higher sodium intake from processed meat can also contribute to elevated blood pressure^33^, reduced arterial compliance, and augmented vascular stiffness^34^. Furthermore, nitrates and their products may facilitate vascular dysfunction and atherogenesis^35^. Thus, based upon the above findings and our results, the reduction of stroke risk may be an effect from higher long chain n-3 PUFA intake through greater ingestion of fish combined with a lower intake of red meat and processed meat intake.

Of these fourteen prospective studies, one study^13^ reported that fish oil supplements were not included in the assessment of dietary intake long chain n-3 PUFAs, and the use of fish oil supplements had little effects on the results. Another study^14^ conducted from 1980 to 1994 reported a fish soil supplement consumption rate of only 1.6% in 1990. Another study^16^ that found no association between fish oil intake and stroke risk reported that approximately 2.7% of participants used fish oil supplements. Two studies^12^,^19^ reported no baseline data on fish oil supplementation, but fish oil supplement use was not common among the participants. The remaining nine studies^9^,^10^,^11^,^15^,^17^,^18^,^20^,^21^,^22^ failed to report any information on fish oil supplement use among the participants. However, eleven studies^10^,^11^,^12^,^13^,^14^,^15^,^16^,^18^,^19^,^20^,^21^ included in this meta-analysis indicated that fish and seafood were the main sources of long chain n-3 PUFAs, while three studies^9^,^17^,^22^ did not specify the sources of long chain n-3 PUFAs. Thus, based upon the above findings, fish and seafood (as opposed to fish oil supplements) were the primary sources of long chain n-3 PUFAs for the participants in the included studies in this meta-analysis.

The stratification results demonstrated that higher long chain n-3 PUFA intake is inversely associated with fatal stroke risk but failed to demonstrate any relationship between ethnicity, stroke type, follow-up duration, or study quality and reduced stroke risk with higher dietary long chain n-3 PUFAs intake. Sensitivity analysis demonstrated a persistent relationship between higher long chain n-3 PUFA intake and reduced stroke risk. Moreover, there was no publication bias detected between the included studies.

Although there is some evidence^36^ suggesting that BMI is a risk factor for stroke, BMI’s influence on the relationship between long chain n-3 PUFA intake and stroke risk remains unknown. In the current stratification analysis, there was a lack of canonical standards for defining BMI subgroups, because this meta-analysis included both East Asian and non-East Asian studies. Specifically, the upper limit for normal BMI in East Asian populations should be 23 kg/m^2^, while the 1997 World Health Organization (WHO) guidelines specify an upper normal BMI limit of 25 kg/m^2^.^37^ Thus, in the stratification analysis for BMI, we applied the midpoint of 24 kg/m^2^ as the cut-off point for BMI (24). Our results showed that individuals with a low BMI (<24 kg/m^2^) demonstrated reduced stroke risk through a higher intake of long chain n-3 PUFAs.

Sex has previously been shown as a factor influencing stroke risk^38^; based on our results, it seems that females benefit more from the increased intake of long chain n-3 PUFAs. As platelets play a pivotal role in development of cardiovascular disease^39^, and platelet aggregation is an early event in the induction of thrombosis and arteriosclerosis^40^, one possible explanation for this sex-based phenomenon may be the differential sex-based effects of anti-platelet aggregation produced by different categories of long chain n-3 PUFAs. There is evidence^41^ showing that DHA, DPA, and EPA are all equally effective in platelet aggregation in females, while both DHA and DPA are significantly less effective in reducing platelet aggregation in males as compared with females (EPA is equally effective in reducing platelet aggregation in both sexes).

There are several notable limitations to this study. First, dietary long chain n-3 PUFA intake tends to be associated with other nutrients that may prevent stroke, such as potassium^42^, magnesium^43^, fiber^44^, and protein^45^. However, the association between long chain n-3 PUFA intake and stroke risk was persistent when we confined the analysis to studies that adjusted for these risk factors. Second, there existed heterogeneity between the included studies, although we are unable to determine the sources of heterogeneity. Third, a healthy diet for the primary prevention of cardiovascular and cerebrovascular disease should include adequate intake of vegetables and fruits^46^,^47^,^48^,^49^ as well as whole grains^50^,^51^ and olive oil^52^.

In conclusion, this meta-analysis reveals that higher long chain n-3 PUFA intake is inversely associated with risk of stroke morbidity and mortality with BMI and sex as key factors influencing this risk. Individuals should be encouraged to manage their body weight while increasing their intake of long chain n-3 PUFAs.

## Methods

### Data sources and searches

PubMed, Embase, Web of Knowledge, and Google Scholar were searched without language restrictions as follows: (“fat” OR “fatty acids”) AND (“stroke” OR “cerebrovascular disease” OR “cerebrovascular disorder” OR “cerebrovascular accident” OR “TIA” OR “transient ischemic attack”). Other potential studies were identified by consulting previous reviews and reference lists of retrieved records.

### Inclusion and exclusion criteria

The inclusion criteria were as follows: (i) a prospective cohort design; (ii) reported RRs and their corresponding 95% CIs for long chain n-3 PUFA intake and stroke risk; (iii) multivariates (such as age, smoking, etc.) were controlled; and (iv) only the most recent publication, or the one with the longest follow-up period, was included when duplicate reports based on the same cohort were used.

The exclusion criteria were as follows: (i) case-control or non-prospective cohort study design; (ii) reviews; (iii) experimental studies; and (iv) conference abstracts.

### Data extraction and quality assessment

Data were extracted independently by two investigators (P.F.C. and W.H.), and any differences were resolved by discussion with a third investigator (X.F.Z.) We retrieved the following parameters from each included study: first author’s name, publication year, country of study population, age range or mean age, sex (%), number of participants, fat intake assessment, follow-up duration, number of stroke events, outcome assessment, RRs of stroke and corresponding 95% CIs for specific fat intake, and covariates adjusted in the statistical analysis. We used the Newcastle-Ottawa Scale (NOS)^23^ to assess the study quality in this meta-analysis with a high-quality study defined as a study with >8 awarded stars.

### Statistical analysis

Log RRs of the highest versus the lowest for cohort studies were weighed by the inverse variance method to obtain pooled RRs. For calculating more robust RRs of stroke from long chain n-3 PUFAs intake, we retrieved all supplemental files of the included studies for RRs of specific types of stroke or specific sex of stroke patient if available. However, 7 of 14 of the included studies did not provide results for males and females separately. For these studies, we contacted the authors to ask them to provide these data. Authors of one study^10^ provided us with separate results for females and males, authors of another study^11^ claimed that they did not have these data, authors of three studies^17^,^18^,^19^ did not respond us, authors of two studies^13^,^20^ were failed to be contacted because of unsuccessful emails and missing contact information; thus, these six studies^11^,^13^,^17^,^18^,^19^,^20^ were not included in the sex subgroup analysis. Stratification analyses were based on BMI (<24 versus ≥24), follow-up duration (<14 versus ≥14 years), ethnicity (non-East Asians versus East Asians), sex (males versus females), stroke type (ischemic versus hemorrhagic), fatal stroke risk, maximum multivariates (pooling RRs of included studies with hypertension, diabetes, and smoking controlled simultaneously), study quality score (≤8 versus >8). For the purpose of obtaining more conservative results, we used a random-effects model for pooling RRs. Smoking, hypertension, and diabetes could not be simultaneously adjusted in one^11^ of the included studies; therefore, we did not include this study in the maximum multivariates adjusted analysis. A meta-regression model was used to detect potential heterogeneity between the included studies. A sensitivity analysis was conducted using the leave-one-out method. Furthermore, publication bias was assessed using Egger’s test. Data obtained from the included studies were analyzed using Stata, version 12.0 (Stata Corp, College Station, Texas).

## Additional Information

**How to cite this article**: Cheng, P. *et al.* BMI Affects the Relationship between Long Chain N-3 Polyunsaturated Fatty Acid Intake and Stroke Risk: a Meta-Analysis. *Sci. Rep.* **5**, 14161; doi: 10.1038/srep14161 (2015).

## Figures and Tables

**Table 1:** Baseline Characteristics of Included Studies.

Number	First author	Year	Country	Age range (mean age)	Sex	No. of participants	Long chain n-3 PUFA intake assessment	Average follow-up (yrs)	Stroke events	Fatal or non-fatal strokes	Maximum adjustment available
1	Morris	1995	USA	40–84	Male	21,185	FFQ	4	173	Both	Age, aspirin and beta-carotene assignment, smoking, alcohol consumption, obesity, diabetes mellitus, vigorous exercise, parental history of myocardial infarction before age 60 years, history of hypertension, history of hypercholesterolemia, vitamin supplement use, and saturated fat intake.
2	Iso	2001	USA	34–59 (<60)	Female	79,839	FFQ	14	574	Both	Age, smoking, time interval, joules, BMI, alcohol, menopausal status, hormone use, exercise, aspirin use, multivitamin use, hypertension, fruit, vegetable, SF, TUF, linoleic acid, animal protein, calcium.
3	Yuan	2001	China	45–64 (56)	Male	18,244	24-hour recall	12	480	Fatal	Age, energy, education, BMI, smoking, alcohol, diabetes, hypertension.
4	He	2002	USA	40–75 (53)	Male	43,671	FFQ	12	608	Both	Age, smoking, BMI, physical activity, hypertension, aspirin use, fish oil, multivitamins, intake of total calories, total fat, saturated fat, trans-unsaturated fat, alcohol, potassium, and magnesium; fruits and vegetable; and hypercholesterolemia.
5	Iso	2003	Japan	40–69 (<60)	Both	4,775	24-hour recall	14.3	295	Both	Age, sex, total energy intake and BMI, hypertension, diabetes, serum total cholesterol, smoking, ethanol intake, and menopausal status.
6	Kaushik	2008	Australia	>49 (65)	Both	2,683	FFQ	12	69	Fatal	Age, gender, blood pressure, BMI, smoking, qualification level, self-rated health, myocardial infarction and stroke.
7	Yamagishi	2008	Japan	40–79 (56)	Both	57,972	FFQ	12.7	972	Fatal	Age, gender, hypertension and diabetes, smoking, alcohol, BMI, mental stress, walking, sports, education, total energy, dietary intakes of cholesterol, saturated and n-6 PUFA, vegetables, and fruit.
8	Montonen	2009	Finland	45–59 (53)	Both	3,958	FFQ	28	659	Both	Age, sex, energy intake, smoking, BMI, physical activity, geographic area, occupation, diabetes, use of post-menopausal hormones, hypertension, serum cholesterol, and consumptions of butter, vegetables, fruits, and berries.
9	de Goede	2012	Netherland	20–65 (41)	Both	20,069	FFQ	10.5	221	Both	Age, smoking, BMI, education, myocardial infarction, alcohol, energy, dietary fiber, vitamin C, beta-carotene, saturated fatty acids, trans fatty acids, monounsaturated fatty acids, linoleic acid, and alpha-linolenic acid.
10	Larsson	2012	Sweden	49–83 (≥60)	Female	34,670	FFQ	10.4	1,680	Both	Age, smoking, education, BMI, physical activity, hypertension, diabetes, aspirin use, myocardial infarction, alcohol, protein, dietary fiber, specific types of fat and cholesterol.
11	Wallstrom	2012	Sweden	44–73 (58)	Both	20,674	FFQ	13.5	755	Both	Age, method version, energy, season, BMI class, smoking, education, alcohol, SBP, antihypertensive treatment, antihyperlipidemic treatment, leisure time physical activity, and energy-adjusted dietary fiber.
12	Koh	2013	Singapore	45–74 (56)	Both	63,257	FFQ	11.1	1,298	Fatal	Age, sex, dialect, year of interview, education, BMI, physical activity, smoking, alcohol, diabetes, hypertension, coronary heart disease, stroke, energy, protein, dietary fiber, saturated fat, monounsaturated fat, omega-6 fatty acids, and alternate omega-3 fatty acids.
13	Takata	2013	China	40–74 (54)	Both	134,296	FFQ and 24-hour recall	5.6, 11.2	864	Fatal	Age, energy, income, occupation, education, comorbidity index, physical activity, red meat, poultry, and vegetable, fruit intake, smoking (men), and alcohol (men).
14	Miyagawa	2014	Japan	(50)	Both	9,190	FFQ	21.2	417	Fatal	Age and sex, smoking, drinking, SBP, blood glucose, serum total cholesterol, BMI, antihypertensive medication use, and residential area. Energy-adjusted intakes of saturated fatty acids, total n-6 PUFA, vegetable protein, total dietary fiber, and sodium.

NA, not available; SBP, systolic blood pressure; BMI, body mass index; SF, saturated fat; TUF, trans-unsaturated fat; PUFA, polyunsaturated fatty acid. FFQ, food frequency questionnaire.

**Table 2:** Long Chain N-3 Polyunsaturated Fatty Acids Intake and Stroke Risk.

Outcome	Studies (N)	Events	Participants	RR	95% CI		*P value*	I^2^for heterogeneity
Total Stroke	14	9065	514483	0.87	0.80	0.95	0.002*	15.1
Fatal stroke risk	6	4228	285642	0.84	0.73	0.97	0.018*	31.3
BMI								
<24	6	4326	287734	0.86	0.75	0.98	0.019*	17.3
≥24	4	2629	61380	0.83	0.68	1.02	0.075	40.2
Follow years								
≤14 years	9	5822	353464	0.87	0.78	0.98	0.026*	24.8
>14 years	5	3243	161019	0.87	0.76	0.99	0.03*	5.4
Race								
Non East-Asians	8	4739	226749	0.86	0.75	0.97	0.049*	21.9
East-Asians	6	4326	287734	0.86	0.75	0.98	0.019*	17.3
Sex								
Female	6	NA	176751	0.81	0.71	0.92	0.002*	12.8
Male	7	NA	131085	0.96	0.84	1.11	0.601	0
Stroke type								
Ischemic	9	4149	416334	0.87	0.76	0.99	0.029*	16.7
Hemorrhagic	8	1551	379250	0.82	0.68	0.99	0.044*	0
Max variates adjusted	13	8201	380187	0.89	0.82	0.96	0.003*	0
Quality								
Score ≤8	7	2855	266805	0.79	0.66	0.94	0.01*	35.7
Score >8	7	6210	247678	0.9	0.82	0.98	0.021*	15.1
